# The balance between intrinsic and ecological fitness reveals hidden regimes in eco-evolutionary population dynamics

**DOI:** 10.21203/rs.3.rs-7403028/v1

**Published:** 2025-09-08

**Authors:** Rowan J. Barker-Clarke, Jason M. Gray, Sydney Leither, Maximilian A. R. Strobl, Jeff Maltas, Dagim Shiferaw Tadele, Michael Hinczewski, Jacob G. Scott

**Affiliations:** 1 Cleveland Clinic Research, Cleveland Clinic, Cleveland, OH, 44106, USA; 2 Department of Physics, Case Western Reserve University, Cleveland, OH, 44106, USA; 3 Department of Computer Science and Engineering, Michigan State University, East Lansing, MI, 48864, USA; 4 Department of Medical Genetics, Oslo University Hospital, Ullevål, Oslo, Norway; 5 Case Western Reserve University School of Medicine, Cleveland, OH, 44106, USA

## Abstract

Understanding how populations evolve requires accounting for both intrinsic fitness, defined by genotype and environment, and ecological interactions that emerge in mixed communities. While evolutionary experiments typically assess fitness in isolation, such monoculture measures may misrepresent dynamics in realistic, interacting populations. Here, we present a game-theoretic framework that explicitly separates intrinsic and ecological contributions to fitness, allowing us to map how ecological interactions can mask, mirror, maintain, or mimic selection driven by genetic differences. We derive analytical conditions for these regimes using deterministic replicator dynamics and validate them in stochastic Wright-Fisher models with mutation and drift. Applying our model to published microbial and cancer co-culture data, we show that real systems span both intrinsic-dominant and ecology-dominant regimes, with ecological effects sometimes reversing or neutralizing intrinsic fitness advantages. These results expose a critical blind spot in experimental design and interpretation, emphasizing the need to account for ecological interactions when inferring evolutionary dynamics and designing therapeutic strategies.

## Introduction

Evolution is often described through the lens of intrinsic fitness, the ability of a genotype to survive and reproduce in a given environment. [1] This idea underpins the powerful concept of the fitness landscape, a driving framework for understanding intrinsic fitness, selection, and evolution. [2–6] Within the peaks and valleys of the fitness landscape, the trajectory of evolution for a population is driven by mutational supply and the relative intrinsic fitness of accessible genotypes. [7–10] The fitness landscape does not account for ecology, yet from cancer cell populations to microbes, evolutionary outcomes are shaped not just by genotype, but by interactions within complex communities.

Ecological and game theoretical modeling approaches such as the Lotka-Volterra and replicator equations are well-established descriptions of how interactions impact the long-term dynamics and stable states of mixtures of interacting populations. [11–14] However, it is the interplay between these two approaches (genotypic evolution and ecology) that is of particular interest in the treatment of evolving, multicellular diseases like cancer and complex infections. For example, while the heterogeneity of genotypes within a patient’s disease is accepted as a driver of both pre-existing or *de-novo* resistance evolution, the presence of cell-cell interactions in this mixed population may further contribute to altering the efficacy of drugs. It is often the case that personalized treatment in evolving diseases eventually encounters resistance, the reasons for which likely include a combination of stochastic, ecological, and genotypic factors. [15, 16]

**Figure 1.**
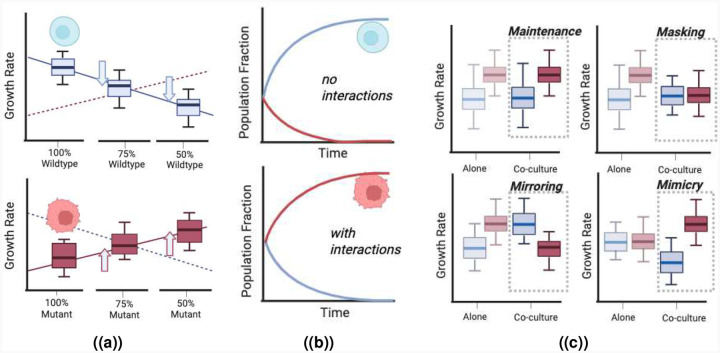
Intrinsic fitness differences combined with ecology can result in counter-intuitive outcomes. Given the monoculture growth rates of two cell types, the null hypothesis is that the same rank and relative growth rate would be sustained. Ecology is often considered to be the deviation from this. Intrinsic fitness differences can be observed by comparing the growth rates of two different populations in monoculture. If the mutant has a higher growth rate than the wildtype in coculture, the mutant outcompetes the wildtype when growing in coculture. (**A**) It is common for experimental scientists to detect ecological interactions by measuring the change in the growth rates of populations in 50:50 co-culture compared to their monoculture growth rates. Frequency dependence can be seen explicitly when ecological effects increase in the presence of increasing fractions of the second type. (**B**) For the example in (**A**) where the mutant has a lower intrinsic fitness than the wildtype, without interactions, the wildtype would outcompete the mutant (**top**). (**C**) Given the importance of intrinsic fitness differences in evolutionary biology, we propose that specific classes can modify the resultant fitnesses in co-culture to result in different evolutionary dynamics and different steady states. We define four representative ways in which interactions confound monoculture expectations. Maintenance, where the original rank and relative distance are preserved. Masking, where original fitness differences are neutralised. Mirroring, where the intrinsic growth rate difference is reversed, and mimicry, where there is no intrinsic fitness difference but one population outgrows another in co-culture.

In recent years, ecological influence on fitness has been directly observed in disease contexts and shown to be a mechanism for maintaining diversity, particularly in bacterial systems. [17–24] Frequency-dependent selection has even been found in cancer cell lines with varying microenvironments: for example, Kaznatcheev *et al*. [24] demonstrated how alectinib-resistant and parental non-small cell lung cancer cells have different fitnesses across relative population frequencies and how the presence of fibroblasts or changing treatment results in distinct frequency-dependent functional changes in fitness (often termed evolutionary games). One way in which ecological interactions can be measured in co-culture experiments is to find the growth rate of each population at a range of starting fractions (Fig 1A). Changes in growth rates due to ecological effects are observed in a dose-dependent way within co-culture and can result in altered evolutionary outcome (Fig. 1B).

We propose four plausible classes of ecological interactions, defined by how ecological factors alter evolutionary outcomes relative to expectations based solely on intrinsic fitness differences (Fig. 1C). Consider two genotypes in each plot, where the red has a higher *intrinsic* fitness than the blue. The first scenario, which we call *maintenance*, occurs when the interactions do not alter the outcome of evolution: a co-cultured mixture of two types will behave exactly as expected from monoculture, the red eventually out-competing blue. This could be due to the absence of interactions, but as we will show below, there is also a set of non-zero interaction strengths that lead to maintenance. The second scenario is *masking*: two genotypes behave as if equally fit (neutral) when co-cultured, despite monoculture fitness differences. The third scenario is *mirroring*: where one is expected to dominate based on monoculture fitness (i.e., red versus blue), but the monoculture expectations are inverted in co-culture (blue outcompetes red to the same degree). The final scenario is *mimicry*: in which two genotypes have equal monoculture fitness but effectively behave as if one has a selective advantage when co-cultured. We focus on these four scenarios because they present interesting examples that clearly illustrate the potential confounding effects of ecology; interactions can be present even if a net effect is absent, and switching between monoculture and co-culture can either exacerbate or mitigate selective differences.

Many papers have also used game theory to model tumor growth and composition, with the presence of interactions between cells, including competing tumor and stromal cells, and the production of growth factors as a strategy. [25–31] Theoretical studies have also shown the potential of frequency-dependent selection to promote high mutation rates and accelerate evolution. [32, 33] Advancing upon prior formulations of frequency-dependent Wright-Fisher models [34, 35] and established co-evolutionary frameworks [36–38], we describe the four scenarios (Fig. 1C) mathematically. Consistent with prior models, we assume that cellular strategies are genetically encoded and inherited, such that payoffs in the evolutionary game represent a cell’s capacity to divide. Like these models, we assume that a cell’s strategy is determined by its genotype and inherited from its parent, such that the payoff in the evolutionary game reflects a cell’s ability to produce identical offspring.

Motivated by increasing empirical evidence, we introduce a biologically grounded game-theoretic framework that explicitly distinguishes between cell-intrinsic and ecological contributions to fitness. This formalism enables the analysis of populations with varying intrinsic growth rates under both deterministic and stochastic dynamics. Notably, the majority of assays in experimental evolution still measure and interpret the fitness of evolving populations primarily as an intrinsic property, independent of ecological interactions.

We identify analytical conditions under which ecological interactions modulate the evolutionary dynamics to preserve, invert, amplify, or obscure the influence of intrinsic fitness differences. Our findings underscore the necessity of integrating ecological interactions into evolutionary models, demonstrating that frequency-dependent selection can significantly reshape evolutionary trajectories. By analyzing payoff matrices from eight published microbial and cancer co-culture experiments [15, 24, 39], we observe both intrinsic-fitness-dominated and ecology-dominated regimes. Notably, ecological effects can shift with environmental context, either reinforcing or counteracting intrinsic fitness differences. These results highlight the importance of jointly considering ecological interactions and genetic fitness landscapes in evolution. These important findings have significant implications in interpreting experiments, designing treatments, and advancing work to control resistance evolution. For example, the application of control theory to steer populations across fitness landscapes must certainly account for the possibility that interactions influence the measured selection landscape. [40] Equally, omitting these factors could limit the success of approaches such as adaptive therapy, which involve mathematical optimization of tumor response with tailored on- and off- drug scheduling. [31, 41]

## Results

In the following, we will model the eco-evolutionary dynamics of two populations: a wild-type population and a mutant population, using evolutionary game theory. We will study the dynamics and steady states of the *proportion $x_{m}=x$* of the mutant population (sometimes denoted the allele fraction). We will use the replicator equation formalism and the game-theoretical payoff matrix to define frequency-dependent fitness and selection.

### Cell-intrinsic and frequency-dependent components can be logically separated within the payoff matrix

We ask, initially, how the traditional payoff matrix from game theory can be expressed in terms of the intrinsic selection coefficient? By decomposing the pay-off matrix into the contributions of cell intrinsic fitness and interaction effects, we can study the impact that each have on the long-term evolutionary outcomes. To decompose the parameters describing eco-evolution in the game theory setting, we use the intuition that for there to be no frequency-dependence of the growth rates, each row of the payoff matrix must be constant, such that the payoff (growth rate) doesn’t depend on the populations present (Box 1).

Consequently, when the interaction terms approach zero, $\left(\alpha_{wm},\alpha_{mw}\to 0\right)$, there are no ecological interactions and no frequency dependence. Additionally, when analyzing these dynamics, we have set the wild-type growth rate $\left(g_{w}\right)$ to 1. As such, there is no explicit time dependence in the resulting dynamics. Should the explicit temporal scale be of interest to the reader, it is straightforward to reintroduce the scaling factor and recover this.

We then use this parameter transformation to rewrite the replicator equation. This equation describes the change in the mutant proportion (x∈[0,1]), not absolute population size, over time. [42] In this parameterisation, the dynamics and evolutionary outcomes (stationary state(s)) of competition between two populations can be written as functions of $\alpha_{wm}$, $\alpha_{mw}$, and $s_{m}$. In this model, any specific evolutionary trajectory depends on the initial condition (the starting mutant fraction, $x_{0}$), the stationary solutions and their stability, and the sign of $\frac{dx}{dt}\left(x_{0}\right)$.

**Figure 2.**
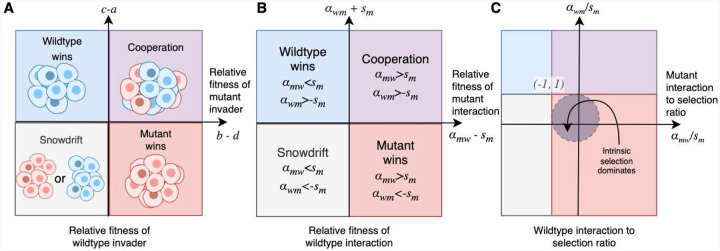
Resultant classes of evolutionary dynamics in the 2-player replicator game as a function of relative fitness and interaction strength. **(A)** Illustration of game dynamics between two populations (wild-type and mutant cells). Evolutionary game theory between two players results in four types of dynamics. The game space reflects distinct evolutionarily stable states corresponding to the four quadrants in relative fitness space (b-d,c-a). In the biological case, both the cell-intrinsic and ecological influences on growth rate come together in co-culture to produce novel dynamics. The observed dynamics rely not only on the isolated behaviour of cells but also on the precise balance between intrinsic and extrinsic factors. In the top left quadrant, the wild type dominates. In the top right quadrant, a heterogeneous mixture is promoted. In the bottom right quadrant, the resistant mutant dominates. Lastly, in the bottom left quadrant, coexistence is unstable, and when the population is not exactly at the unstable fixed point (coexistence fraction), the population is driven to the nearest stable fixed point (all wild types or all mutants). (**B**) In the cell-centered decoupled payoff framework, the (b-d,c-a) axes and conditions on the game parameters for each quadrant are rewritten in terms of the intrinsic fitness difference (selection coefficient) and the interaction coefficients. (**C**) In the interaction-selection plot, a system's position is plotted as a function of interaction strengths $\left(\alpha_{ij}\right)$ divided by the intrinsic selection coefficient $s_{m}$. The coordinates in our mutant and wild-type system are $\left(\alpha_{mw}/s_{m},\alpha_{wm}/s_{m}\right)$. The regions are labeled under the assumption that $s_{m}>0$ and the mutant is fitter than the wild type. We highlight the unit circle in this space as the region where intrinsic selection is stronger than interaction. In this region, the fitter monoculture population (in this example, the fitter population is the mutant with $s_{m}>0$) remains dominant. The color of the quadrants and position of the boundaries in this plot refer to four different universality classes within deterministic evolutionary games. Boundaries between the classes in all phase spaces occur along the lines $\alpha_{wm}=-s_{m}$ and $\alpha_{mw}=s_{m}$ and thus are mirrored accordingly in the interaction-selection plot depending on its sign.

To further visualize the relative contributions of interaction terms and intrinsic fitness differences, we introduce the “interaction-selection” plot (Fig. 2), in which the axes are the ratios of extrinsic vs intrinsic fitness of the wildtype and mutant type, respectively $\left(\alpha_{mw}/s_{m},\alpha_{mw}/s_{m}\right)$. In this representation, the unit circle is the region where intrinsic selection differences are dominant, whereas outside this region, ecological interactions are the dominant contribution to the fitness of the population. To understand how these components combine to influence the long-term dynamics we overlay the long-term steady-state onto this plot (corresponding to the four quadrants of the traditional game-space plot). In this way, we can see that when ecological contributions are small (ratio of interaction to selection is inside the unit circle), the mutant population wins due to its higher assumed intrinsic fitness. However, as we change the strength and direction of ecological interactions, all four evolutionary scenarios are achievable, *despite the intrinsic fitness difference remaining unchanged*. The boundaries between the universality classes in all phase spaces occur along the lines $\alpha_{wm}=-s_{m}$ and $\alpha_{mw}=s_{m}$ For example, the cooperation quadrant (top right) is defined by $\alpha_{wm}>s_{m}$ and $\alpha_{mw}>-s_{m}$. In this way, we can see that as we vary the difference in intrinsic fitness $\left(s_{m}\right)$, the quadrants of different evolutionary outcomes are translated along the second diagonal and overlaps exactly with the primary axes when $s_{m}=0$.

Biologically, while intrinsic selection advantages can be present, cells within heterogeneous populations must survive selection under eco-evolutionary dynamics. Taking into account the effects of both ecological and mutational forces, survival of or competitive exclusion by a sub-population may play out in a variety of ways: the maintenance of existing intrinsic selection advantages, the masking of selection differences, or the mimicry of intrinsic selection in its absence. Furthermore, one can also observe mirroring, the complete inversion of selection (direction and magnitude) due to interactions. These four interaction classes are of interest when interpreting the results of co-evolutionary dynamics in experiments.

### Game-modification of the intrinsic selection coefficient

**Figure 3.**
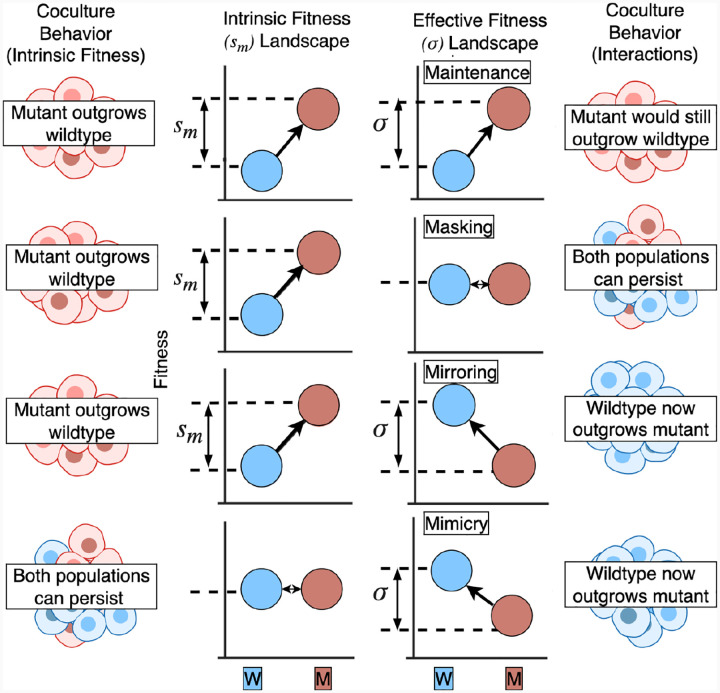
Effective fitness landscapes and definitions of maintenance, masking, mirroring, and mimicry in terms of the selection coefficient. There exists an intrinsic monoculture fitness landscape representing the relative fitness difference $\left(s_{m}\right)$ of two different cell types (**left**), with two genotypes (wildtype and mutant) represented and an arrow from the lower to higher fitness genotype. Under this selection coefficient or fitness difference, there is a baseline expectation of how the evolutionary dynamics of a mixed population (blue and red) would play out: the proportion of the fittest genotype in the population increases towards 100%. For example, if the mutant (red) has a higher fitness in the effective fitness landscape, the population should be dominated by mutants. For co-cultured populations, interactions can modify the fitnesses and result in different evolutionary dynamics in co-culture. This effective fitness landscape has a new selection difference of σ. Thus, the evolutionary outcomes are modified by interactions, and the four rows show four representative ways in which interactions confound monoculture expectations: maintenance, masking, mirroring, and mimicry.

Ecological dynamics, as described in game theory, between wild-type and mutant alleles result in frequency-dependent selection with a dependence on the intrinsic growth rates of the cells and the interactions $\alpha_{wm}$ and $\alpha_{mw}$. We hypothesized that the four interaction classes (as illustrated in Fig. 3) are the result of specific relationships between the interaction-dependent selection and interaction-independent intrinsic selection coefficient, which are in turn determined by the relationship between the interaction coefficients. To do so, one must ask what form the frequency-dependent selection coefficient would take?

To discuss frequency-dependent dynamics relative to the intrinsic selection coefficient, we derive a new frequency-dependent selection coefficient. To do this we find the frequency-dependent fitness of each allele using the payoff matrix in Eq. (B1). We thus define the frequency-dependent selection coefficient $\sigma_{m}(\vec{x})$ that is to replace the constant selection coefficient $s_{m}$:

(1) $$\sigma_{m}\left(x\right)=\frac{s_{m}+\alpha_{mw}-\left(\alpha_{wm}+\alpha_{mw}\right)x}{1+\alpha_{wm}x}.$$

With this frequency-dependent selection coefficient defined, we can compare intrinsic (non-interacting) selection to frequency-dependent selection across varying intrinsic and interaction strengths. When these interaction terms go to zero $\left(\alpha_{wm},\alpha_{mw}\to 0\right)$, this reduces to the intrinsic selection coefficient, $s_{m}$.

### Systems with non-trivial interactions can reproduce frequency-independent dynamics

It is important to note that when there are no ecological interaction terms $\left(\alpha_{wm},\alpha_{mw}\to 0\right)$, the replicator equation reduces such that there are only two steady-state solutions, $x_{\infty }\in \{0,1\}$. This is frequency-independent deterministic evolution. When selection, $s_{m}$, is non-zero, one population eventually outcompetes the other, the mutant proportion becoming x=1 (100%) or x=0 (0%) depending on the sign of $s_{m}$.

In this replicator equation framework, we can define the concepts of maintenance, mirroring, masking, and mimicry via the preservation or inversion of the selection coefficient. In all of these cases, frequency dependence is removed despite the presence of non-zero interaction terms. As such, these solutions result in identical dynamics to the reference non-interacting case across time for all starting fractions. The alteration of the effective fitness landscapes through interactions, producing maintenance, masking, mimicry, and mirroring, are illustrated in Figure 3.

Maintenance occurs when the frequency-dependent selection coefficient is kept constant, $\sigma_{m}(x)=s_{m}$. In this context, the interaction terms are non-zero but leave the evolutionary outcome unchanged, producing the same modal equilibrium value as if there were no interactions. In the second regime, fitness differences between a wild-type and a mutant in monoculture may be counteracted by interactions when they are grown in co-culture. We call this the *masking* scenario, where intrinsic fitness differences are neutralized by interactions. Masking occurs when the frequency-dependent selection coefficient, $\sigma_{m}\left(x,\alpha_{mw},\alpha_{wm}\right)=0$. The reverse case is also possible, where wild types and mutants may have a neutral fitness relationship in monoculture, but when grown in co-culture, there is a selective fitness difference. Mimicry occurs when there is a zero intrinsic selection coefficient $\left(s_{m}=0\right)$, but a non-zero frequency-dependent selection coefficient, $\sigma_{m}(x)=s^{\prime }$. Mirroring occurs when the direction and magnitude of the selection coefficient are inverted during frequency-dependent selection, $\sigma_{m}(x)=-s_{m}$.

When these conditions upon the frequency-dependent *selection coefficient* occur, the resultant dynamics are indistinguishable from the respective frequency-independent mapping. These regimes and the restrictions they place on the interaction coefficients are summarised as follows,

(2) $$\begin{array}{cc} \text{DYNAMICS} & \\ \text{Maintenance}:\sigma_{m}(x)=s_{m} & \to \alpha_{mw}=\alpha_{wm}=0\\ \text{Mirroring}:\sigma_{m}(x)=-s_{m} & \to \alpha_{mw}=-2s_{m},\alpha_{wm}=2s_{m}/\left(1-s_{m}\right)\\ \text{Masking}:\sigma_{m}(x)=0 & \to \alpha_{mw}=-s_{m},\alpha_{wm}=s_{m}\\ \text{Mimicry}:\sigma_{m}(x)=s^{\prime } & \to \alpha_{mw}=s^{\prime },\alpha_{wm}=-s^{\prime }\left(1+s^{\prime }\right). \end{array}$$

The full derivations of the deterministic selection coefficients can be found in the Supplementary Information, Section S3. Within the four cases defined, it is important to emphasize that the only possible *maintenance* is the trivial solution, $\alpha_{mw}=\alpha_{wm}=0$. Across the three other non-trivial cases, the deterministic dynamics of the interacting system are indistinguishable from those of a non-interacting reference system, independent of starting conditions. Thus, when observing the dynamics of a system, it is possible for dynamics consistent with a non-interacting system of intrinsic selection coefficient $s_{m}$, to be produced by an interacting system.

### Interacting systems can reproduce non-interacting equilibrium solutions

When we observe systems, for example, in experiment or *in vivo*, we are not always able to observe the full *temporal dynamics* of a system, and effective selection is sometimes inferred from the steady state of a system. Having shown that we can define our interaction classes using the equivalence of the replicator equations, it is also possible to define these regimes via the equivalence of evolutionary outcomes (stationary solutions). We ask the question: for what (less strict) parameter ranges do deterministic eco-evolutionary systems with interactions reproduce steady-state solutions that are indistinguishable from possible outcomes of systems without interactions?

In the presence of game interactions, stationary solutions for the mutant fraction (x) in systems obeying the replicator equation can be defined by game coefficients $\alpha_{wm}$, $\alpha_{mw}$, and the mutant selection coefficient $s_{m}$. For the steady-state solution, $x_{\infty }^{\text{int}}$, to the deterministic replicator equation with interactions, we can define *maintenance* and *mirroring* as dependent on the sign of $s_{m}$, which determines the stable fixed point in the non-interacting system.

A definition of *masking* requires the production of the same steady state as that obtained when $s_{m}=\alpha_{mw}=\alpha_{wm}=0$, but the replicator equation is identically zero in this case and thus *masking* is difficult to define in the deterministic system by steady state alone. Mimicry requires that the steady state be equivalent to the steady state for a non-interacting system with selection coefficient $s^{\prime }$, which requires one interaction coefficient to be zero, dependent upon the sign of $s^{\prime }$. If $s_{m}=0$ and both of the coefficients are non-zero, a steady state other than $x_{\infty }=0,1$ will be produced.

(3) $$\begin{array}{cc} \text{STEADY}\;\text{STATE} & \\ \text{Maintenance}:x_{\infty }^{int}=x_{\infty }^{s_{m}} & \to \alpha_{wm}=\text{sgn}\left(s_{m}\right)s_{m}\\ \text{Mirroring}:x_{\infty }^{int}=1-x_{\infty }^{s_{m}} & \to \alpha_{mw}=-\text{sgn}\left(s_{m}\right)s_{m}\\ \text{Masking}:x_{\infty }^{int}=x_{\infty }^{0} & \to \alpha_{mw}=\text{Not}\;\text{defined}\\ \text{Alternate}\;\text{Masking}:x_{\infty }^{int}=\frac{1}{2} & \to \alpha_{mw}=\alpha_{wm}-2s_{m}\\ \text{Mimicry}:x_{\infty }^{int}=x_{\infty }^{s^{\prime }} & \to \alpha_{mw}=0\;\text{or}\;\alpha_{wm}=0. \end{array}$$

Thus, maintenance, mirroring, and mimicry of the steady state, for the deterministic replicator equation, are definitionally *indistinguishable* from the steady-state solution of the non-interacting system, and masking has a reasonable, non-trivial equivalence. Further, the parameter constraints for *maintenance* and *mirroring* are only partially constrained, with one of the interaction coefficients being only constrained by the exclusion set $\alpha_{wm}\neq -\alpha_{mw}$. For example, the same steady-state outcome (e.g., fixation of the mutant population) can result from a non-interacting system with unique interaction parameters and non-unique selection coefficient $\left(\alpha_{wm}=\alpha_{mw}=0,s_{m}>0\right)$ or be the result of maintenance, where a non-unique set of interacting systems satisfy relaxed constraints of $\alpha_{mw}=s_{m}$ and $\alpha_{wm}\neq -\alpha_{mw}$.

### Defining a frequency-dependent selection-coefficient for the Fokker-Planck model

The reality in biology is that evolutionary dynamics (with or without interactions) are *stochastic*, and noise is introduced through stochastic processes such as measurement, genetic drift, and mutation. Noise can significantly alter the dynamics and steady states of systems. It is unknown, from both the mathematical and experimental perspectives, how noise impacts our ability to distinguish interacting and non-interacting systems. To begin to address this question, we extend our approach to a mathematical model of ecology and evolution that includes stochasticity via genetic drift and mutation. We use a partial differential equation to model the time evolution of the probability density function of the mutant fraction; the form we choose is known as the Fokker-Planck equation (see Box 2 for details). [43, 44]

The decomposition of the payoff matrix (Eq. (B1)) highlights how these terms are interdependent in replicator systems and how the deterministic outcomes (game quadrants) depend on the interaction and selection coefficient. Further, we have outlined how the balance between the interaction and selection coefficients can result in maintenance, mirroring, masking, and mimicry (Eq. (2)). To understand the possibility that these interaction classes occur in stochastic systems, we must define the selection coefficient in the stochastic model. To do so, we analyze how the probability density distribution of the mutant fraction, the solution to the partial differential equation, shifts with different interaction strengths. To define the four interaction classes, we first express the Fokker-Planck stationary solution in terms of the selection and interaction terms $\left(s,\alpha_{wm},\alpha_{mw}\right)$. In particular, our concepts of maintenance, masking, mirroring, and mimicry rely on how the modal value of this solution shifts relative to the mode of the probability density distribution for the system *without* interactions.

In the modification of the population by ecological interactions, our specific cases of interest reflect the position of the modal value of the population with respect to the initial modal value of a system without interactions (Eq. (B5)). However, while numerical calculation of the modal value for given interaction coefficients and intrinsic selection is possible (e.g., Fig. S2), a general expression for the peak of the distribution with interactions is intractable.

As an alternative analytical approach to gain insight into the effects of game interactions, we examined the selection coefficient $\sigma_{m}(x)$ from Eq. (1) evaluated at the modal frequency $\left(x_{\text{mode}}\right)$ in the absence of interactions. This can be interpreted as the hypothetical selection effect that would be observed if we were to suddenly “turn on” interactions in a previously non-interacting system. It provides information about how the system would evolve in response to these interactions and allows us to define the same eco-evolutionary regimes described in the next section. The expression for $\sigma_{m}\left(x_{\text{mode}}\right)$ is given by:

(4) $$\sigma_{m}\left(x_{\text{mode}}\right)=\frac{2s_{m}^{2}+s_{m}\left(\alpha_{mw}-\alpha_{wm}\right)+\left(\text{sgn}\left(s_{m}\right)2\mu -\sqrt{4\mu^{2}+s_{m}^{2}}\right)\left(\alpha_{mw}+\alpha_{wm}\right)}{2s_{m}+\alpha_{wm}\left(s_{m}+\sqrt{4\mu^{2}+s_{m}^{2}}-\text{sgn}\left(s_{m}\right)2\mu \right)}.$$

We see that when there are no ecological interaction terms $\left(\alpha_{wm},\alpha_{mw}\to 0\right)$ this expression for the frequency-dependent selection coefficient recovers the non-interacting result, $\sigma_{m}(x)\to s_{m}$. It is therefore the case that for a given frequency-dependent selection coefficient $\sigma =\sigma_{m}\left(x_{\text{mode}}\right)$, there is a fixed relationship between the interaction coefficients $\alpha_{mw}$ and $\alpha_{wm}$. This can be found by rearranging Eq. (4).

### Maintenance, masking, mimicry, and mirroring can occur in the stochastic Fokker-Planck model of ecology and evolution

To investigate the previously described interaction classes, we can use our formula for the stochastic frequency-dependent selection coefficient $\sigma_{m}\left(x_{\text{mode}}\right)$. We use the conditions on the selection coefficient, as described for the deterministic system, to obtain simplified expressions that define the four regimes, summarized in Eq. 5.

The first regime, *maintenance*, is defined in the Fokker-Planck model, as $\sigma_{m}\left(x_{\text{mode}}\right)=s_{m}$. Non-trivial maintenance is only possible via the effects of mutations, since when μ→0 the regime reduces to the trivial non-interacting case $\alpha_{wm},\alpha_{mw}=0$. *Masking*, is defined in the stochastic model as $\sigma_{m}\left(x_{\text{mode}}\right)=0$ when $s_{m}\neq 0$. At $x_{\text{mode}}$, we can express *mimicry* as $\sigma_{m}\left(x_{\text{mode}}\right)=\sigma \neq 0$, a constant, when $s_{m}=0$. In this stochastic model, *mirroring* is defined as $\sigma_{m}\left(x_{\text{mode}}\right)=-s_{m}$.

In mimicry and maintenance, we find that the results include a term of first order in μ added to the deterministic result for each regime. As such, the limit μ→0 corresponds to regimes that are present in the previously described deterministic systems. In the mirroring case, an extra term of order $\mu^{-1}$ is present. The graphical representation of these relationships is illustrated for a range of fixed seletion $\left(s_{m}\right)$ and mutation (μ) parameters (Fig. S5).

(5) $$\begin{array}{cc} \text{SELECTION}\;\text{AT}\;\text{EQUILIBRIUM} & \\ \sigma >0 & \sigma <0\\ \text{Maintenance}:\alpha_{wm}=\frac{\mu \alpha_{mw}}{s_{m}\left(1+s_{m}\right)} & \alpha_{mw}=-\frac{\mu \alpha_{wm}\left(1+s_{m}\right)}{s_{m}}\\ \text{Mirroring}:\alpha_{wm}=-\frac{s_{m}\left(2s_{m}+\alpha_{mw}\right)}{\left(s_{m}-1\right)\mu }-\frac{2s_{m}}{\left(s_{m}-1\right)}+\frac{2s_{m}+\alpha_{mw}}{\left(s_{m}-1\right)s_{m}}\mu & \alpha_{mw}=\frac{s_{m}\left(-\alpha_{wm}+s_{m}\left(2+\alpha_{wm}\right)\right)}{\mu }-2s_{m}+\frac{2s_{m}-\alpha_{wm}\left(1-s_{m}\right)}{s_{m}}\mu \\ \text{Masking}:\alpha_{wm}=\alpha_{mw}+2s_{m} & \alpha_{mw}=\alpha_{wm}-2s_{m}\\ \text{Mimicry}:\alpha_{wm}=-\frac{\sigma }{1+\sigma }+\frac{\mu \left(\alpha_{mw}-\sigma \right)}{\sigma (1+\sigma )} & \alpha_{mw}=\sigma -\frac{\mu \left(\sigma +(1+\sigma )\alpha_{wm}\right)}{\sigma } \end{array}$$

The surfaces described in Eq. (5) define the maintenance, masking, mimicry, and mirroring regimes. These regimes are defined by constraints on one interaction coefficient as a function of the other system parameters. Thus each condition is a surface in three (for masking) or four-dimensional space (for the other regimes). In the no mutation limit (μ→0) we see that we recover conditions for the equivalent regimes in deterministic systems.

**Figure 4.**
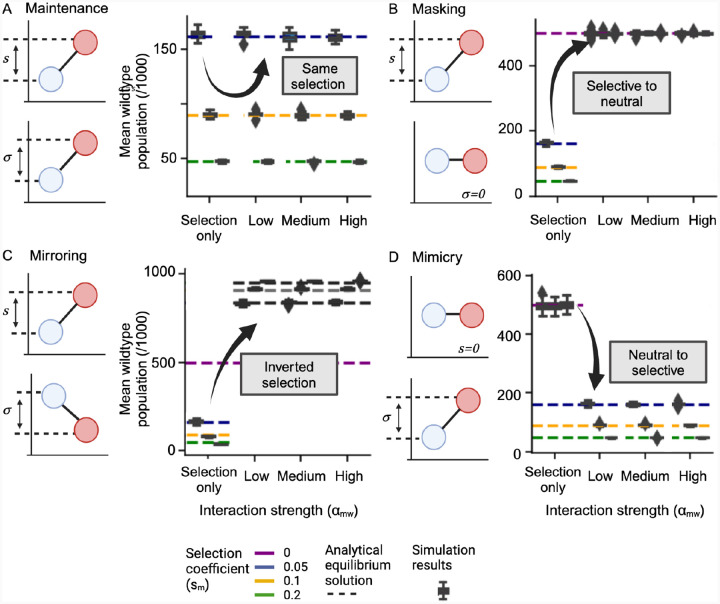
Analytical conditions for the four interaction classes (Maintenance, Masking, Mirroring, and Mimicry) are validated in stochastic simulations and validity is insensitive to initialized selection and interaction strength. Simulations were carried out for each scenario and a genetic population comprising individuals with one of two possible alleles (N=1000), with no interactions (Selection only), and with interactions. Dashed lines represent the theoretical modal value of the wild-type fraction at different intrinsic selection values. For all scenarios, we compare non-ecological simulations with interaction coefficients → 0 (Selection only), with simulations with increasing strength of ecological interactions. Added ecological interactions have the ratios defined in Eq. (5). (**A**) For maintenance, the simulations maintain the same mean wild-type fraction as in the case with no interactions. (**B**) For the masking scenario, the initial modal fraction of the wild type at each selection coefficient is not 50%. When interactions are introduced, the modal population changes to 50%, the equivalent of no selection. (**C**) In the case of mirroring, the population fraction for three different selection coefficients and interaction coefficients align with the analytical model. (**D**) In the mimicry case, the initial population is 50% as the mutant population has no intrinsic selection coefficient. With the mimicry interactions, we recapitulate the specific selection coefficients in each case. All simulations show agreement with our theoretical conditions across different strengths of $\alpha_{mw}$ and different values of s and μ. Mean population values are averaged over 3000 generations after an initial 1000 generations. Each box plot represents the distribution of 50 independent simulations. Initial conditions for the simulations were a 50:50 wild-type (0) and mutant (1) population.

To validate the theoretical restrictions (on $\alpha_{wm}$ and $\alpha_{mw}$) that result in the regimes discussed above, we simulated the dynamics of an evolving population with and without the interaction terms (Fig 4). The plots at the top of this figure display the relationships from Eq. (5) for each example case. We show boxplots of simulation results, where initial simulations with no interactions (“Selection only”) are compared to simulations with added interaction terms for three different intrinsic selection coefficients and two mutation rates.

In the *maintenance* case, the no-interaction simulations fall on the theoretical fractions (dotted lines) for the appropriate selection $\left(s_{m}\right)$ as expected. As predicted theoretically, the mean populations in the *maintenance* interaction cases remain centered on the same theoretical value as in the no interactions (“Selection only”). The population means have the same values. Changing ecological interactions maintain the intrinsic selection.

In the *masking* case, the non-interacting simulation populations align with the theoretical predictions for a given intrinsic fitness difference favoring the mutant. As also predicted theoretically, under *masking*, this intrinsic selection difference is nullified by ecological interactions. The mean population value after *masking* is centered on the equal 50:50 split.

In the *mirroring* case, the no-interaction cases are aligned with the analytical results expected for the given selective differences. As predicted theoretically, the mean populations in the *mirroring* case are all perfectly reflected, and the model value moves to the inverted fraction (1-x) of the population. Thus the selection advantage of one population is inverted by the ecological interaction.

In the *mimicry* case, the theoretical modal values for all simulations with no interactions are at 50% as marked with the purple dotted line. The no-interaction cases are all neutral as expected in the mimicry scenario. As predicted theoretically, the mean populations in the *mimicry* case all lie on the theoretical means for the three selection coefficients.

### Ecological interactions can produce maintenance, masking, and mirroring effects in *in vitro* experiments

**Figure 5.**
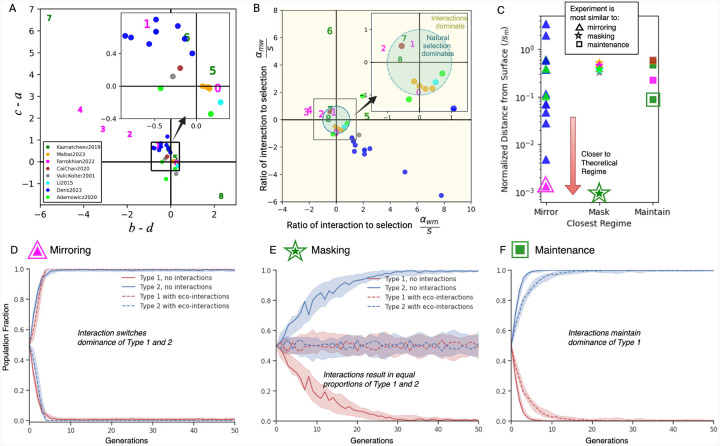
Interaction strengths decoupled from intrinsic selection shows differential dominance of ecology and experimental evidence of potentially misleading ecologically dominated regimes. Games, colored by publication reference, as measured in published experiments. Farrokhian (0–4) and Kaznatcheev (5–9) experiments are numbered for communication and illustration purposes. **A**) Experimental measured games are displayed in “traditional” relative growth rate encoded game space. Inset plot expands area for visibility. **B**) Interaction-selection plot. Distribution of published experimental interaction values relative to intrinsic selection. The unit circle (blue) is the boundary representing equal magnitudes of ecological and intrinsic selection. Experiments inside the circle are dominated by intrinsic selection and those outside the circle are dominated by interaction effects. **C**) Distances of published payoff composition from theoretical maintenance, masking, and mirroring surfaces are shown. The experimental payoff matrices closest to masking (green star), maintenance (blue square) and mirroring (pink triangle) are highlighted. **D-F**) Simulations using the payoff matrices from the highlighted experiments in (**C**) illustrate the time-dependent eco-evolutionary dynamics. The population dynamics are simulated for the three experiments: most mirror-like, masking-like, and maintenance-like.

To demonstrate how the reformulation of the payoff matrix can help ecologists, game theorists, and biologists to understand the range of possible eco-evolutionary dynamics that may produce certain experimental outcomes, we carried out a literature search to identify directly measured payoff matrices or co-culture experiments from which the payoff matrix [24] can be defined (see Methods). The traditional game-space representation of the payoff matrices from the literature is shown in Fig 5A. The color represents the publication from which the payoff matrix is obtained, and each dot is an individual reported measurement of the payoff (or growth rates). Numbered experiments represent different payoff results from the same paper and are numbered to highlight specific ordering and positional changes between plots. While this traditional plot is sufficient to determine the proportions of populations at equilibrium, the plot is non-unique with respect to the intrinsic and extrinsic contributions to the payoff matrix. We used our payoff matrix reformulation and examined published results with sufficient information to determine the magnitudes of selection and interaction effects. [15, 16, 24, 39, 46–48] We show the interaction-selection plot, presenting the ecological interaction coefficients $\left(\alpha_{mw},\alpha_{wm}\right)$, on the axes, divided by the cell-intrinsic selection coefficient $s_{m}$. This new interaction-selection plot illustrates the direction and intensity of the two interaction terms in a measured payoff matrix. Within this plot, the unit circle (blue) shown in Fig. 5B represents the boundary between dominance of ecological versus intrinsic selection effects. Within this circle, a system's dynamics are dominated by intrinsic fitness differences. Outside of the circle, the dynamics are dominated by ecological effects. We see that this visualization gives us additional information, including allowing us to distinguish strong and weak ecological effects relative to selection that may result in very similar equilibrium proportions (i.e. point 1 and 6). This plot allows us to see the relative strengths of individual eco-effects compared to selection. For example, result 7, with large positive c-a value and large negative b-d value in Fig. 5A lies close to the unit circle in Fig. 5B. The distribution of values from Deris *et al*. [48] all have strong reported ecological effects (negative, competitive) relative to selection differences. We see that for Farrokhian *et al*. [15](pink, 0–4), the relative strength of interactions is higher in 3 than 4 (Fig. 5B) and the sign of the interaction effect on the mutant is reversed in the no-drug condition (0)(Fig. 5B). We note that all bacteria interaction terms lie within [–2,2]. The two most extreme examples of interaction-to-selection ratios $\frac{\alpha_{ij}}{s_{j}}>5$ are in cancer models (6, cell line [24] and dark blue, mouse models [48] respectively).

We have previously presented the concepts of maintenance and masking to highlight that when ecological interaction effects are present in co-culture, they can reinforce prior assumptions. In assuming that there is an absence of interaction or an absence of intrinsic selection, it is possible to miss potentially confounding biological components underlying a similar net result. To understand to what extent the specific regimes of maintenance, masking, and mirroring may be present in real multi-species systems, we examine the experimental coordinates of the selection and interaction coefficients and their position with respect to the aforementioned theoretical regimes.

Each of the *maintenance, masking*, and *mirroring* regimes is defined by a function $a_{wm}=f\left(s_{m},a_{mw},\mu \right)$ (Eq. (5)). These conditions thus correspond to analytically defined manifolds. The minimum distance of the experimental system from each theoretical regime was calculated as the perpendicular distance from the measured value $\left[s_{m},a_{mw},a_{wm}\right]$ to the closest projection of the point onto the surface. These values are shown for each paper and each condition in Fig. 5C. Multiple systems are seen as approximating maintenance (star), mirroring (circle), and masking (triangle), where experiments closer to the theoretical regimes are closer to the x-axis. The experiment most closely matched to each interaction class is highlighted. One of the experiments from Farrokhian *et al*. (pink, triangle) is closest to mirroring, and two of the Kaznatcheev *et al*. experiments (green, star, square) are closest to masking and maintenance, respectively. In Fig. 5D–F we simulate the eco-evolutionary dynamics of the populations using the published parameters for the highlighted experiments. Dynamics are plotted without the interaction components (solid lines) and then when the interactions are present (dashed lines). While the Kaznatcheev experiment in **F** still maintains approximately the same selection coefficient, it deviates from exact maintenance, particularly in contrast to the alignment to mirroring and masking dynamics in **D** and **E**.

For proximity to maintenance and mirroring surfaces, we approximate a fixed μ=0.001 for the experiments comparing growth rates of populations accessible by mutation in monoculture and coculture. *Mimicry* was excluded from this analysis because, although the presentation of a selective advantage from purely ecological means is an interesting evolutionary mode/adaptation, testing for it requires prior knowledge of the otherwise arbitrary “target” selection coefficient σ.

Key details about the publications and data points are shown in Table 1, and the complete data table is included in the Supplementary Information (Table S2). For each publication, either the directly reported payoff matrix or a payoff derived from the published growth rates in monoculture and coculture were used (see Methods). It is worth noting that the Cai et al. paper [39] uses metabolic models to determine a numerical payoff matrix. Separately, in Deris *et al*. [48], the calculations of the payoff matrices (dark blue, triangles) are averaged over several replicates within ten different types of mice, each with Lewis Lung Carcinoma (LLC). Additionally, the method of calculation in Deris *et al*. sets the payoff matrix term b to zero, setting $\alpha_{wm}=-1$ (see Box 1, Eq. (B1)). This is why the Deris *et al*. experiments most closely approximate mirroring.

In summary, we derive a general expression for the equilibrium distribution of a population obeying the Fokker-Planck equation with selection and arbitrary interactions. We reveal, using analytical theory and simulations, the potential impacts of game interactions and their ability to completely alter evolutionary dynamics. We find the critical boundaries at which these game dynamics either maintain a population at the originally fitter genotype (maintenance), move a population from the fixation of the fitter genotype in monoculture to the fixation of the other (mirroring), promote fixation of a population in the absence of intrinsic selection differences (mimicry), or even promote the heterogeneity of a population by leveling the playing field (masking).

We find that the ability for interactions in co-culture to maintain the same relative monoculture fitness advantage depends on the mutation rate between the two populations. Additionally, the interaction strengths required to mask selection and promote neutral evolution and co-existence are independent of the mutation rate. Finally, we find that equally fit populations in monoculture can interact in ways in co-culture to perfectly mimic the composition resulting from the co-evolution of genotypically distinct populations. This result means that the measured outcome of any mixed population, such as tumor cells, must be interpreted with caution.

## Discussion

The approach of the experimental biologist often requires the isolation of microscopic populations to understand their properties. In contrast, ecologists focus primarily on the interactions of populations on a macroscopic scale. While both agree that interactions between populations can have implications, they work under assumptions often at extreme ends of the spectrum. We focus on the eco-evolutionary regime in which both intrinsic fitness and ecological interactions play a role. We also focus on a framework that attempts to discuss the conditions under which the dynamics, or steady state, of an interacting population may be indistinguishable from the possible outcomes of non-interacting ones.

These ideas find importance in the ongoing study of human diseases, where *in vitro* work has also solidified the existence of cell-cell interactions and their importance. Experimental scientists using “game assays” have also begun to quantify these interactions between cells in culture. [24] Understanding how to bridge the gap between co-evolution in complex disease and theory is critical to future treatment approaches. We use ecology and game theoretical concepts to model and explore the possible impacts of these interactions as they become increasingly important in the disease and cell-biology perspectives. [49]

Despite this growing experimental evidence of frequency-dependent interactions in microbial and cancer systems, most evolutionary studies still treat fitness as a fixed, context-independent property. By reframing the game matrix such that the interactions and monoculture fitnesses are separated, we come to a form that can be interpreted within the Fokker-Planck formalism but also better understood from the perspective of evolutionary population dynamics in cell lines. Our work builds on existing experimental and mathematical descriptions of eco-evolutionary dynamics. [15, 36–38]

We highlight the critical nature of game interactions from an experimental perspective, demonstrating exactly how the presence of game interactions, in addition to the underlying genotypic fitness difference, has the potential to modify the evolutionary outcome of an evolving population to any desired outcome. Even in our simplified case, the (observed) fitness of the cells in question can be significantly altered by moderate cell-cell interactions. We posit that growth dynamics in mixed populations may readily bear little resemblance to monoculture growth rates and that initial seeding (e.g., experimental or metastatic) ratios may have strong impacts on resultant dynamics. Without specific techniques designed to robustly assay frequency-dependent game interactions, [15, 24] the magnitude and impact of ecological interactions cannot be quantified. These effects have widespread potential ramifications, for example, when assaying chemotherapeutic drugs in isolated cell lines, when developing cancer cell lines *ex vivo*, and when interpreting evidence for neutral evolution in tumors. [50–54] We reviewed the experimental literature for payoff matrices, bringing together a range of data in a single framework via our novel interaction-selection plot examining interaction effects. We also demonstrated the relevance of these potentially counter-intuitive eco-evolutionary modes in published data. Further work aimed at outlining to what extent these modes of eco-evolution can be seen in systems with logistic growth and carrying capacities, typically modeled using Lotka-Volterra competition models, [12] would allow further application of these concepts to additional experiment types.

Having shown that deterministic game theory is significantly limited in distinguishing the outcomes of interacting and non-interacting systems, we have only begun to explore the potential to distinguish these outcomes within stochastic frameworks. While we do show that our stochastic analytical and simulation results are in agreement, we have not approached the reverse problem, the inference of game parameters from the simulation results. This is an important next step, as this stochastic description of eco-evolutionary dynamics over time may allow us to leverage larger quantities of inherently stochastic experimental results to infer interaction properties. Experimentalists frequently collect observations across multiple replicates and time-series, but are limited in their ability to utilize this data in current frameworks for assessing co-culture systems.

Further limitations of this modeling approach include the non-mechanistic formulation of game interactions and the assumed linearity of the frequency-dependent interactions, an assumption that likely does not hold true across all systems. Additionally, to analyze and discuss previously published results from the literature, simplifying assumptions were made about mutation rates. Although in the published results we see several relatively strong ecological effects, the results shown are not exhaustive. Although interactions here are evident, there is likely a positive bias in the literature toward the publication of experimental results where ecological interactions are present. Whilst our work focused on the modal value of the equilibrium distribution after a long period, the varying higher-order properties of the Fokker-Planck distributions suggest that systems with games may also have altered evolutionary dynamics over time. This has been noted in previous work, [33] where Kaznatcheev demonstrated that evolution is, in some cases, accelerated by game interactions. Future work could derive explicit expressions for signatures of game dynamics that are encapsulated in the higher-order properties (standard deviation, skew) of the equilibrium solutions. Identifying these factors may be critical in interpreting the existence and strength of cell-cell interactions in experimental populations. Decomposition of the payoff matrix provides a biologically meaningful formulation of the payoff matrix and the ability to independently modify the cell-intrinsic and ecological contributions to the growth rate. In addition to supplying a new modeling paradigm and analytical solution in the case of added noise, this formalism provides an ideal starting point for deeper analysis of experimental results in the context of intrinsic selection and stochasticity. This model framework also more readily permits the integrated modeling of evolutionary therapy, permitting the direct mapping between monoculture and co-culture results and a stochastic mathematical description of an evolving population. [6, 40] These frameworks can assist calculations for optimal stochastic control and adaptive therapy in the presence of experimentally motivated drug and micro-environmental-dependent ecological cell interactions. [15, 41, 55–58]

## Materials and Methods

### Theory

To develop our theoretical approach, we build on a simple model of evolutionary game dynamics in the absence of noise and mutation. Game theory, in general, is the study of the dynamics that result from the interaction of different strategies played against each other. Specific strategies result in expected payoffs for the players, and thus the average payoff depends upon the frequency of strategies in the system. Differential game theory can describe deterministic solutions for these dynamics using differential equations. We assume symmetry whereby the payoffs in a 2-strategy symmetric differential game can be presented as a payoff matrix in the following way [14, 59]:

(6) $$P=\left(\begin{array}{ll} a & b\\ c & d \end{array}\right),$$

where a is the payoff for a player with strategy 1 playing against another player of type 1, b is the payoff for a player with strategy 1 playing against a player of type 2, c is the payoff for a player with strategy 2 playing against a player of type 1, and d is the payoff for a player with strategy 2 playing against a player of type 2. These values determine the expected payoffs or “fitnesses” for each player when the frequencies of strategies in the population are known.

In evolutionary models, available “strategies” equate to the cell type as a cell does not choose, but inherits its strategy; the strategy (phenotype) and identity (genotype) form a one-to-one mapping. Under the assumption that a cell's genome determines its strategy, we use a simplified model comprising a single locus with two alleles, the wild-type and the mutant, the latter harboring a resistance mutation at the site of interest. In genetic population models, strategy proportions change when cells undergo self-replication, with fitter strategies reproducing at a faster rate.

The underlying assumption of replicator dynamics is that the fitness of a cell with strategy i is proportional to the difference between the fitness of its strategy and the average. This determines its rate of replication and this formalism and accompanying equations are referred to as replicator dynamics. [13, 59] In the 2-dimensional case with payoff matrix P from Eq. (6), the replicator equation takes the following form:

(7) $$\frac{\partial x}{\partial t}=x\left(1-x\right)\left(\left(c-a\right)\left(1-x\right)-\left(b-d\right)x\right),$$

where the proportion of type 2 (mutant) is x, and thus the proportion of type 1 (wild type) is 1-x. Without the addition of noise, the replicator equation gives us deterministic solutions for the evolutionarily stable strategies present. [60] Without mutation in the population, this equation has stationary solutions at x=0, x=1, and x=(c-a)/((b-d)+(c-a)). Following this, different relative magnitudes of a, b, c, and d result in various evolutionarily stable solutions. One can construct the 2-dimensional “game space”, which has the axes c-a and b-d as shown in Figure 2. In the top left c>a and d>b, the wild-type always outgrows the mutant. In the bottom right the converse is true. In the top right, c>a but b>d, so the species co-exist. In the bottom left c<a and b<d such that there is an unstable mixed equilibrium that cannot persist within a stochastic system. A specific two-player game payoff matrix has a position in one of these quadrants that categorizes it into a certain universality class. Games in the same quadrant generate similar dynamics. Different quadrants are often named in the literature as a class of game, e.g., Prisoner's Dilemma (top left), Snow Drift (bottom left), Stag Hunt(bottom right), and Harmony (top right).

While the derivations for the deterministic conditions of the different regimes is outlined in the Supplementary Information, S3, the full mathematical derivation for the stochastic systems is outlined in the accompanying Mathematica notebook file.

### Simulations

Using Python (version 3.11.5), we developed a stochastic Wright-Fisher type population model with discrete non-overlapping generations modified from Bedford *et al*. [61]. Creating a two-genotype model, we incorporated generational sampling that was weighted by frequency in ratios given by the coefficients of a payoff matrix. We used this to observe the simulated evolutionary trajectories and the stationary distribution before and after the addition of game interactions of different strengths. In particular, we asked whether our simulation results are consistent with game interactions predicted theoretically to maintain, mimic, mirror, or mask the expected evolutionary outcome that would be based on monoculture fitness alone.

The simulations (parameter ranges in Table 2), involved a constant population of size N comprised of two species, denoted wild-type ('0') and mutant ('1'), undergoing mutation (rate μ) and selection at each generation. The sampling frequency of each population was based on the frequency-dependent fitness of each population calculated at each generation. The constant population size N, mutation rate μ, and mutant advantage $s_{m}$ were all fixed for each simulation. When random net payoff matrix values or interaction strengths $\left(\alpha_{ij}\right)$ were required, they were generated using a random uniform distribution within a given range. In each simulation, populations were allowed to evolve from an initial fraction $x_{i}$ for 1000 generations, at which point the population fraction from each simulation was extracted.

### Intrinsic selection and extrinsic interaction values from the literature

We found coculture experiments published in the literature, [15, 16, 24, 46–48] and converted them to normalized and decoupled payoff matrices. Decoupled payoff matrices are expressed in terms of both intrinsic selection and ecological growth rate effects (see Eq. (B1)). Our search was restricted to published results that explicitly reported experimentally derived payoff matrices [15, 16, 24]or publications with experimental growth rates for populations in both mono- and co-culture. Details of the publications, system or experiment type, and number of payoff matrices are shown in Table S1.

To calculate the payoff matrix and thus interaction values and selection from mono- and co-culture growth rate information, it is possible to construct an ecological interaction (*growth-frequency*) plot for both populations (Fig. S7) from which the intersections (payoff values) can be inferred. With the payoff matrix generated, intrinsic and extrinsic growth rate modifiers can be calculated.

### Distance of experiments from maintenance, masking, and mirroring surfaces

The maintenance, masking, and mirroring surfaces were defined in Eq. (5). To calculate the proximity of the published experiments' payoff values to the theoretical manifolds representing maintenance, masking, and mirroring (see GitHub for code), we used Python to carry out numerical optimization. A small mutation rate was assumed and set at 0.001 for experimentally derived distance calculations.

The unitless Euclidean distance between two points was calculated and minimized with respect to points on the surface to approximate the distance of the measured point from the respective manifold. Numerical optimization (numPy) was used to find the shortest distance between the measured value, $\left(s_{m}^{\text{exp}},\alpha_{wm}^{\text{exp}},\alpha_{wm}^{\text{exp}}\right)$ and points on the surfaces defined for maintenance and mirroring. A Euclidean distance function was minimized subject to this surface constraint. The optimized point is the projection of the original point onto the surface. Then the distance $\left(d_{\text{min}}\right)$ between the experimental point and its projection is calculated numerically. An illustration of this idea in less than four dimensions is in Fig. S8.

## Supplementary Material

This is a list of supplementary files associated with this preprint. Click to download.

- MMMM2025RewriteSI.pdf

## Figures and Tables

**Table 1.** Experimental payoff systems and associated papers. Results from the following papers were used to derive corresponding selection and interaction coefficients in their payoff matrices. *Li *et al*. found that the relationship between frequency and fitness was non-linear, but the linear assumption in our model was used for linear payoff matrix estimation from their data.

Author	Year	System	Variable	Number of matrices
Vulic and Kolter [46]	2001	Bacteria (*E. coli*)	Mutation	1
Li *et al*.* [22]	2015	Bacteria (*Curvibacter*, *Duganella*)	Species	1
Kaznatcheev *et al*. [24]	2019	Non-small cell lung cancer	Drug+CAFs	4
Adamowicz *et al*. [47]	2020	Bacteria (*E. coli* and *S. enterica*)	Mutation	3
Cai *et al*. [39]	2020	In-silico	Metabolic simulation	1
Farrokhian *et al*. [15]	2020	Non-small cell lung cancer	Mutation, Drug	5
Deris *et al*. [48]	2023	Lewis lung cancer (murine)	Mouse types	10
Maltas *et al*. [16]	2024	Non-small cell lung cancer	Mutation, Drug	4

**Table 2.** Methodological parameter values and ranges for the frequency-dependent Wright-Fisher model

Parameter	Symbol	Value(s)
Mutation rate (per generation)	μ	1 × 10^−2^, 1 × 10^−3^
Population size	N	100–10000
Generations	-	500–1000
Initial fraction (i)	$x_{i}$	(0,1)
Intrinsic selection	s	(0,0.5)
Interaction effect of i on j	$\alpha_{ij}$	(−1,1)

## Data Availability

All of the Mathematica and Python scripts in this project can be found on GitHub at; https://github.com/rbarkerclarke/MaskMimicMaintain. The processed previously published experimental data is available in the supplementary files and the same repository.

## Box 1: Deterministic game theoretical framework and the payoff matrix

In the payoff matrix for two distinct cell populations undergoing replicator dynamics, the diagonal terms, a and d from Eq. (6) can be understood as the intrinsic monoculture growth rates of each population. The off-diagonal elements reflect the growth rate of the population in the presence of the other cell type. We model this as the population’s intrinsic growth rate modified by extrinsic cell-cell interactions. To illustrate this clearly, we represent and decompose the classical payoff matrix to show the inter-dependence of coefficients as follows:

(B1)

Here $g_{w}$ and $g_{m}$ are the intrinsic growth rates of the wild-type and mutant cells respectively, $\beta_{wm}$ is the fitness difference of the wild-type in the presence of the mutant population, and $\beta_{mw}$ is the fitness difference of the mutant in the presence of the wild type population. In the second line, we have normalized all terms relative to the wild-type growth rate, defining the mutant selection coefficient $s_{m}=g_{m}/g_{w}-1$ and two interaction coefficients $\alpha_{wm}=\beta_{wm}/g_{w}$, $\alpha_{mw}=\beta_{mw}/g_{w}$.

After this variable transformation, the well-known replicator equation, Eq. (7), for the mutant fraction in our model can be rewritten as follows:

(B2)
$$\frac{dx}{dt}=x\left(1-x\right)\left(\left(\alpha_{mw}+s_{m}\right)\left(1-x\right)-\left(\alpha_{wm}-s_{m}\right)x\right).$$

Using both the dynamics described by this equation and the possible steady state solutions, we define the maintenance, mirroring, masking, and mimicry regimes in the text.

## Box 2: Stochastic formalism

Using a Fokker-Planck description of our system, we denote ρ(x,t) as the probability density function of a population having mutant fraction x at time t. As shown by Kimura, [45] Wright-Fisher dynamics with mutation and selection can be described via the following reaction-diffusion equation,

(B3)
$$\frac{\partial \rho (x,t)}{\partial t}=-\frac{\partial }{\partial x}[v(x)\rho (x,t)]+\frac{\partial^{2}}{\partial x^{2}}[D(x)\rho (x,t)],$$

The first term on the right describes the deterministic effects of mutation and selection, which shift the peak of ρ(x,t) over time, while the second term leads to a diffusive broadening of the distribution due to demographic noise (finite sampling from a population of size N). For a non-interacting system, the expressions D(x) and v(x) in these terms are: [45]

(B4)

where μ is the mutation rate (assumed here to be symmetric for wild-type to mutant and vice versa), $s_{m}$ is the mutant selection coefficient, and g(x)=x(1-x). We have used the frequency-dependent selection coefficient to modify the first term in the Fokker-Planck equation, replacing the selection coefficient, $s_{m}$ in v(x) with the frequency-dependent selection coefficient $\sigma_{m}(x)$. The diffusion term remains unchanged.

### The most likely evolutionary outcome as the mode of the probability density function

The location of the mode (the peak of the probability density distribution) can be determined by setting $\partial \rho^{\text{eq}}(x)/\partial x=0$ (or equivalently ∂U(x)/∂x=0). In the no-interaction limit, $\alpha_{mw},\alpha_{wm}\to 0$, with mutation rate μ and intrinsic selection coefficient $s_{m}$, the peak of the stationary probability distribution is given by the following relation:

(B5)
$$x_{\text{mode}}\left(\mu ,s_{m}\right)=\frac{-2\mu +s_{m}+\text{sgn}\left(s_{m}\right)\sqrt{4\mu^{2}+s_{m}^{2}}}{2s_{m}}.$$

We use the modal population fraction, $x_{\text{mode}}$, the peak of this probability density function, to define equivalent regimes. See Methods and Supplementary Information for further details.
